# Primary Central Nervous System Burkitt Lymphoma With Non-Immunoglobulin Heavy Chain Translocation in Right Ventricle: Case Report

**DOI:** 10.3109/08880018.2011.566599

**Published:** 2011-05-26

**Authors:** Ming Jiang, Jiang Zhu, Yong-song Guan, Li-qun Zou

**Affiliations:** 1 Department of Medical Oncology of Cancer Center, West China Hospital, Sichuan University, Chengdu, P. R. China; 2 Department of Medical Oncology of Cancer Center, West China Hospital, Sichuan University, Chengdu, P. R. China; and State Key Laboratory of Biotherapy West China Hospital, Sichuan University, Chengdu, P. R. China

**Keywords:** Burkitt lymphoma, combination therapy, primary central nervous system Burkitt lymphoma, primary central nervous system lymphoma

## Abstract

Primary central nervous system Burkitt lymphoma (PCNSBL) is rare. Few cases of primary central nervous system involvement with sporadic Burkitt lymphoma have been reported and its treatment is now controversial. Here, the authors report a case of a 14-year-old boy suffering from non-immunoglobulin heavy chain (IgH) translocation PCNSBL. To the authors' knowledge, this is the second case report describing primary Burkitt lymphoma involving cerebral ventricles. After receiving combination treatment with surgery, stereotacticradiosurgery, and a chemotherapy regimen including high-dose methotrexate, the patient had a disease-free survival of 18 months.

Burkitt lymphoma (BL), rare and aggressive, is a form of B-cell non-Hodgkin lymphoma with a tendency to disseminate to the bone marrow and meninges [1]. Central nervous system involvement occurs in about 13% to 17% of adults [2] and 12% of children with BL [3]. However, primary central nervous system Burkitt lymphoma (PCNSBL) is very rare. An extensive search of the literature showed that just a few cases of PCNSBL have been reported in the past 30 years; only one reported that the tumor located in the third ventricle and the left temporal horn [4].

Here, we present a case study about a 14-year-old boy who complained of intermittent blunt headache caused by PCNSBL and had a long-term survival after combination therapy.

## CASE REPORT

**FIGURE 1:**
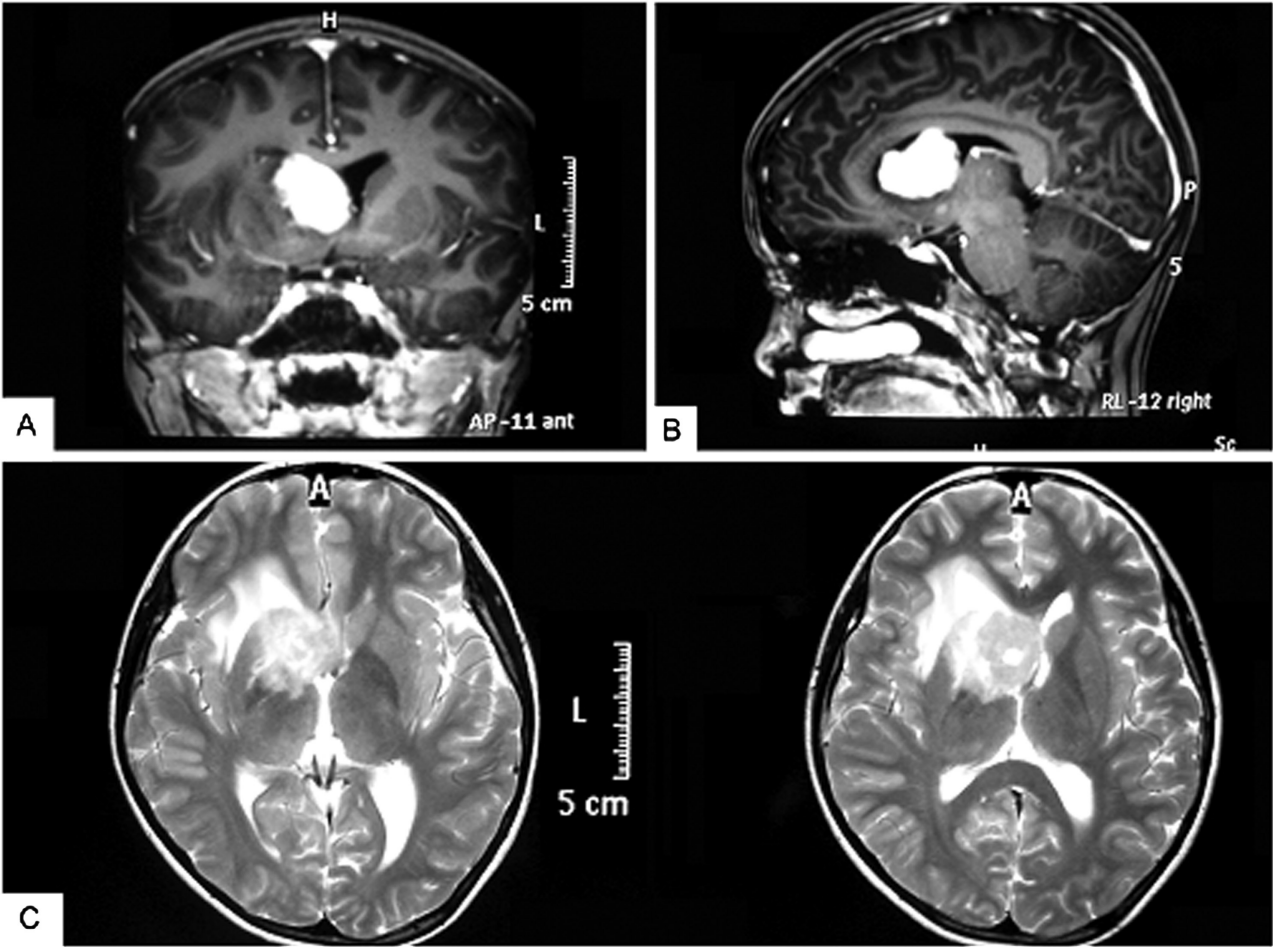
Brain magnetic resonance imaging of the patient before treatment. (A) A 3.4 × 2.3 × 2.4 cm mass in the anterior horn and body of the lateral ventricle with isointensity on the T1-weighted axial image. (B) Sagittal view of the same fragment. (C) Mass with slight edema on the T2-weighted image.

A 14-year-old boy complained of intermittent blunt headache without obvious cause for 2 months. He had no vomiting, myasthenia, or hypoesthesia, no stool and urine problems, and no fever, night sweats, or weight loss. On admission, he was found to be in good physical condition with a Karnofsky performance score of 90, having neither systemic nor neurological signs. Laboratory studies, including routine blood tests, liver and kidney function, and serum lactate dehydrogenase (LDH), showed results within the normal range. Serum tests for virus infections, including those caused by hepatitis B or C, human immunodeficiency virus (HIV), and Epstein-Barr virus (EBV), were negative. Contrast-enhanced magnetic resonance imaging (MRI) of the head detected a mass measuring 3.4 × 2.3 × 2.4 cm in size, isointense on T1- and T2-weighted pulse sequences, affecting the right anterior horn and body of the lateral ventricle with ring enhancement, with slight edema of white matter in the right frontal and parietal lobes, the right basal ganglia, the external capsule, and the callosity, and with left shift of the midline (Figure 1). A giant cell astrocytoma or ependymocytoma was strongly suspected.

**FIGURE 2:**
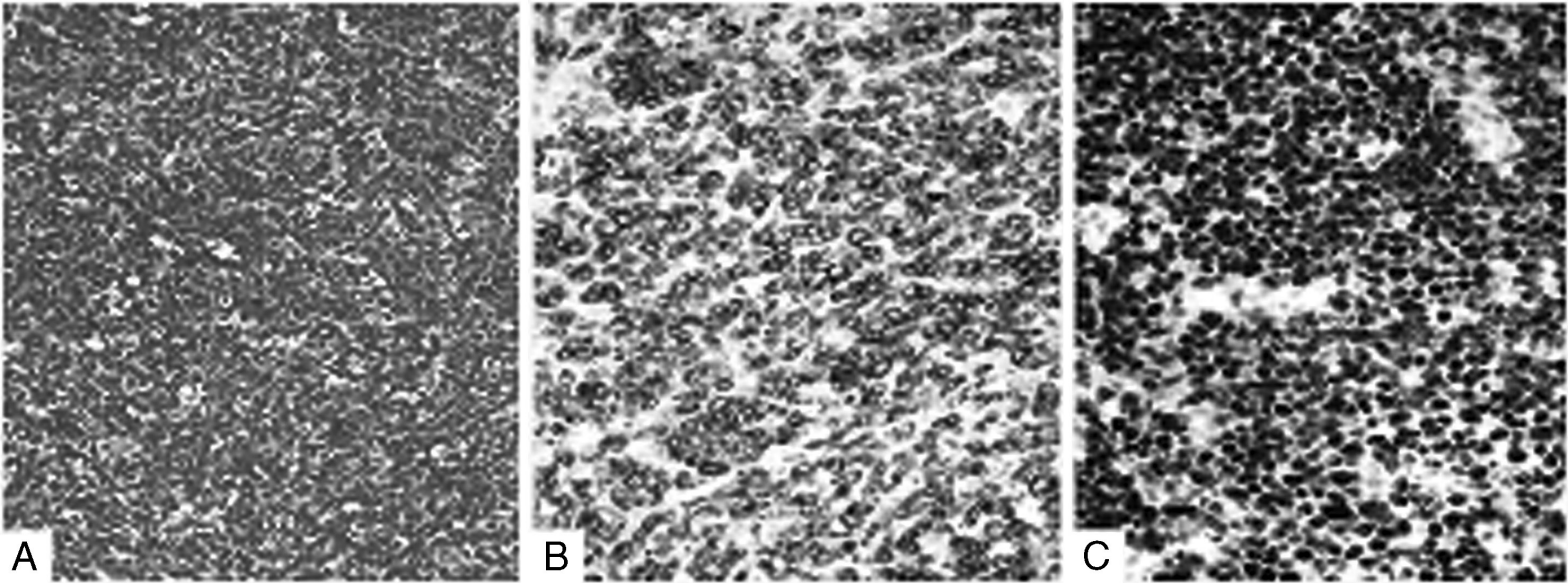
Histology and immunohistochemistry. (A) Tumor tissue showed “starry-sky” appearance with medium-sized lymphocytes. Hematoxylin and eosin (HE) stain, original magnification × 200. (B) Positive CD79a on the membrane of the tumor cell (magnification × 400). (C) More than 95% of cells expressed Ki-67 (magnification × 400).

**FIGURE 3:**
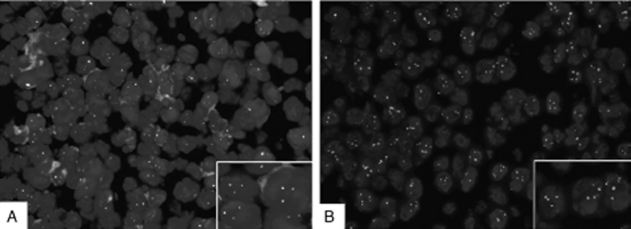
A break-apart probe showed that tumor cells have *c-myc* translocations. (A) Tricolor fusion probe for c-myc/*IgH* showed that tumor cells were negative for *t(8;14),* which (B) indicates that the partner of myc was probably *IgL* or some other gene.

Surgery was preformed in December 2008 in our hospital and revealed a tumor, solid in nature, firm, fish-shaped, and having an affluent blood supply. The tumor, which was located in the right lateral ventricle, laterally closely adherent to the thala-mus, was removed entirely. Postoperative hematoxylin and eosin (HE) staining of the tumor tissue showed a characteristic starry-sky appearance and medium-sized lymphocytes. Immunohistochemistry demonstrated CD20+, CD 10+, CD79a+, BCL-6+, TdT−, MPO−, BCL-2−, CD3−, and Ki-67+ (>95%) (Figure 2). Fluorescent in situ hybridization (FISH) analysis (c-myc dual-color break-apart rearrangement probe; Vy-sis) found that MYC/IGH fusion probe was negative but MYC break-apart probe was positive for the tumor cells (Figure 3). Owing to interventricular foramen adhesion after his surgery, the boy repeatedly had intracranial hypertension, so a ventriculoperi-toneal shunt was inserted 2 days after the initial operation. In order to exclude systemic non-Hodgkin lymphoma, whole-body positron emission tomography/computed tomography (PET/CT) scan, bone marrow biopsy, and testicular ultrasound were performed and no abnormal findings were obtained. Furthermore, spinal MRI and cere-brospinal fluid (CSF) cytological examination yielded normal results. The diagnosis of PCNSBL was established.

After surgery, the symptom of headache disappeared and no nervous disturbances were observed. Gamma-knife therapy was carried out with border dose of 16 Gy, covered by a 45% isodose curve. Later chemotherapy was administered in a 21 -day cycle with a methotrexate (MTX)-based protocol (MTX 3 g/m^2^, day 1, intravenous [i.v.]; vin-cristine 1.4 mg/m^2^, day 1, i.v.; prednisone 100 mg, days 1–5 per os [p.o.]; leucovorin [CF] 15 mg/m^2^, every 6 hours on days 2,3, and 4, i.v.). A total of 6 cycles of chemotherapy were delivered. No grade 2 or higher toxicities were found. Up until now, the patient has been disease-free for 18 months.

## DISCUSSION

Primary central nervous system lymphoma (PCNSL) is an aggressive form of non-Hodgkin lymphoma developing in the brain, spinal cord, eyes, or leptomeninges without evidence of systemic involvement [5]. Overall, PCNSL accounts for 0.5% to 2% of all primary brain tumors and 0.7% to 0.8% of all lymphomas. More than 90% of PCNSLs are histologically classified as high-grade B-cell lymphomas, the remaining cases are T-cell lymphomas (2% to 5%), mucosa-associated lymphoid tissue (MALT) lymphoma, and others [8]. PCNSBL is rare and few cases have been reported [4, 6–18]. It often appears in cerebral hemisphere, basal ganglial, thalamus, and corpus callo-sum lesions [19]. Until now, only one case has been reported to involve the cerebral ventricles [18].

The diagnosis of Burkitt lymphoma was made by typical “starry-sky” appearance in HE stain and immunohistochemical findings according to the World Health Organization classification of tumors of lymphoid tissues [20]. Although most of the cases of BL have c-myc translocations, up to 10% of cases maybe c-myc negative. In about 80% of cases the translocation is between c-myc and the IgH genes, and in the remaining 20% the translocation is between c-myc and the non-IgH genes [21]. In our patient, FISH analysis showed that c-myc/IgH fusion probe was negative but c-myc apart probe was positive for the tumor cells, which implies the translocation is between c-myc and some non-IgH genes.

Intensive short-course chemotherapy combining with intrathecal injection regimen was recommend to treat systemic BL [22], whereas for PCNSL, the high-dose MTX-based chemotherapy regimen has been commonly used, which improves median disease-free and overall survival of up to 30 to 40 months from the survival of 12 to 18 months usually seen after using cranial radiotherapy (CRT) alone [23]. Some studies use CRT plus high-dose MTX as first-line therapy [24], but more researches recommend CRT after some form of initial chemotherapy, especially to patients older than 60 years. Moreover, a pediatric series of PCNSL has reported that immunocom-petent and immunodeficient children with PCNSL may be cured with chemotherapy alone without CRT [25]. So the role and the timing of CRT are intriguing yet controversial.

To date, 14 sporadic PCNBL cases have been reported in the English literature [4, 6–18]. Of the 15 cases, 5 are involving children (including ours) [8, 10, 13, 14], although the treatment regimens were different, including surgery, radiotherapy, and systemic and intrathecal chemotherapy. It seemed that the patients who received a combination management of MTX-based chemotherapy, radiotherapy, and/or surgery [9–11] had much longer overall survival time than did those who only received surgery [6, 12]. However, the best regimens are still under discussion.

Our patient had an isolated brain mass <3.5 cm in diameter at the age of 14 years and his spinal MRI and CSF cytology were negative. Thus we used gamma-knife therapy instead of CRT to decrease the risk of CRT-related neuropsychological sequelae [26]. During the whole 6 cycles of chemotherapy, no grade 2 or higher toxicities were found. Up to now, the tumor has been in complete remission for 18 months and our patient is attending school with no problems.

In summary, PCNSBL is a rare type of non-Hodgkin lymphoma, which has no specific clinical manifestations and imaging features. Treatment is still controversial. Our case report demonstrates that for isolated PCNSBL cases without spread to cerebrospinal fluid, the combination therapy of surgery, gamma-knife, and high-dose MTX-based protocol without intrathecal injection is effective and acceptable for children. This strategy reduced the risk of CRT and intrathecal injection and need to be studied for more details.

## References

[1] Non-Hodgkin's lymphoma, NCCN clinical practice guidelines in oncology, V.1.2010.MS-21 http://www.nccn.org/professionals/physiciangls/PDF/hodgkins.pdf.

[2] Blum KA, Lozanski G, Byrd JC (2004). Adult Burkitt leukemia and lymphoma. Blood..

[3] Cairo MS, Sposto R, Perkins SL (2003). Burkitt's and Burkitt-like lymphoma in children and adolescents: a review of the Children's Cancer Group experience. Br J Haematol..

[4] Ye G, Hou YY, Zhang XB (2010). Primary central nervous system Burkitt lymphoma as concomitant lesions in the third and the left ventricles: a case study and literature review. J Neurooncol..

[5] Batchelor T, Carson K, O'Neill A (2003). Treatment of primary CNS lymphoma with methotrexate and deferred radiotherapy: a report of NABTT 96-07. J Clin Oncol..

[6] Kobayashi H, Sano T, Ii K (1984). Primary Burkitt-type Lymphoma of the central nervous system. ActaNeuropathol (Berl)..

[7] Mizugami T, Mikata A, Hajikano H (1987). Primary spinal epidural Burkitt's lymphoma. Surg Neurol..

[8] Toren A, Mandel M, Shahar E (1994). Primary central nervous system Burkitt's lymphoma presenting as Guillain-Barre syndrome. Med Pediatr Oncol..

[9] Spath-Schwalbe E, Genvresse I, Stein H (1999). Primary cerebral highly-malignant B-cell lymphoma of the Burkitt type. Dtsch Med Wochenschr..

[10] Silfen ME, Garvin JH, Hays AP (2001). Primary central nervous system lymphoma in childhood presenting as progressive *panhypopituitarism*. J Pediatr Hematol Oncol..

[11] Wilkening A, Brack M, Brandis A (2001). Unusual presentation of a primary spinal Burkitt's lymphoma. J Neurol Neurosurg Psychiatry..

[12] Monabati A, Rakei SM, Kumar P (2002). Primary Burkitt lymphoma of the brain in an immunocompe-tent patient. Case report. J Neurosurg..

[13] Shehu BB (2003). Primary central nervous system Burkitt's lymphoma presenting with proptosis. Ann Trop Paediatr..

[14] Daley MF, Partington MD, Kadan-Lottick N (2003). Primary epidural burkitt lymphoma in a child: case presentation and literature review. Pediatr Hematol Oncol..

[15] Gobbato PL (2006). Primary meningeal Burkitt-type lymphoma presenting as the first clinical manifestation of acquired immunodeficiency syndrome. Arq Neuropsiquiatr..

[16] Abel TW, Thompson MA, Kim J (2006). Primary central nervous system Burkitt lymphoma: report of a case confirmed with identification of t(8;14)by fish. Brain Pathol..

[17] Sun J-M, Chen G-J, Ho C-L (2008). Primary Burkitt lymphoma of the CNS in an immunocompetent elderly woman. J Med Sci..

[18] Kozáková D, Macháleková K, Brtko P (2008). Primary B-cell pituitary lymphoma of the Burkitt type: case report of the rare clinic entity with typical clinical presentation. Cas Lek Cesk..

[19] Küker W, Nägele T, Korfel A (2005). Primary central nervous system lymphomas (PCNSL): MRI features at presentation in 100 patients. J Neurooncol..

[20] Swerdlow S, Campo E, Harris NL (2008). WHO Classification of Tumours of Haematopoietic and Lymphoid Tissues.

[21] Ferry JA (2006). Burkitt lymphoma: clinicopathologic features and differential diagnosis. Oncologist..

[22] Mead GM, Sydes MR, Walewski J (2006). An international evaluation of CODOX-M and CODOX-M alternating with IVAC in adult Burkitt's lymphoma: results of United Kingdom Lymphoma Group LY06 study. Ann Oncol..

[23] Abrey LE, Yahalom J, DeAngelis LM (2000). Treatment for primary CNS lymphoma: the next step. J Clin Oncol..

[24] DeAngelis LM, Seiferheld W, Schold SC (2002). Combination chemotherapy and radiotherapy for primary central nervous system lymphoma: radiation Therapy Oncology Group Study 93–10. J Clin Oncol..

[25] Abla O, Sandlund JT, Blaser S (2004). Primary central nervous system lymphoma in children: a report of 12 cases. Blood (ASH Annual Meeting Abstracts).

[26] Batchelor T, Loeffler JS (2006). Primary CNS lymphoma. J Clin Oncol..

